# *M. tuberculosis* Induces Potent Activation of IDO-1, but This Is Not Essential for the Immunological Control of Infection

**DOI:** 10.1371/journal.pone.0037314

**Published:** 2012-05-23

**Authors:** Antje Blumenthal, Gayathri Nagalingam, Jennifer H. Huch, Lara Walker, Gilles J. Guillemin, George A. Smythe, Sabine Ehrt, Warwick J. Britton, Bernadette M. Saunders

**Affiliations:** 1 Department of Microbiology and Immunology, Weill Cornell Medical College, New York, New York, United States of America; 2 The University of Queensland Diamantina Institute, Brisbane, Queensland, Australia; 3 Mycobacterial Research Program, Centenary Institute Sydney, New South Wales, Australia; 4 Department of Pharmacology, School of Medical Sciences, University of New South Wales, Sydney, New South Wales, Australia; 5 Faculty of Medicine, University of New South Wales, Sydney, New South Wales, Australia; 6 Program in Immunology and Microbial Pathogenesis, Weill Graduate School of Medical Sciences of Cornell University, New York, New York, United States of America; 7 Department of Medicine, University of Sydney, Sydney, New South Wales, Australia; Institut Pasteur, France

## Abstract

Indoleamine 2,3-dioxygenesae-1 (IDO-1) catalyses the initial, rate-limiting step in tryptophan metabolism, thereby regulating tryptophan availability and the formation of downstream metabolites, including picolinic and quinolinic acid. We found that *Mycobacterium tuberculosis* infection induced marked upregulation of IDO-1 expression in both human and murine macrophages *in vitro* and in the lungs of mice following aerosol challenge with *M. tuberculosis*. The absence of IDO-1 in dendritic cells enhanced the activation of mycobacteria-specific T cells *in* vitro. Interestingly, IDO-1-deficiency during *M. tuberculosis* infection in mice was not associated with altered mycobacteria-specific T cell responses *in vivo*. The bacterial burden of infected organs, pulmonary inflammatory responses, and survival were also comparable in *M. tuberculosis*-infected IDO-1 deficient and wild type animals. Tryptophan is metabolised into either picolinic acid or quinolinic acid, but only picolinic acid inhibited the growth of *M. tuberculosis in vitro*. By contrast macrophages infected with pathogenic mycobacteria, produced quinolinic, rather than picolinic acid, which did not reduce *M. tuberculosis* growth *in vitro*. Therefore, although *M. tuberculosis* induces robust expression of IDO-1 and activation of tryptophan metabolism, IDO-1-deficiency fails to impact on the immune control and the outcome of the infection in the mouse model of tuberculosis.

## Introduction

A critical requirement for controlling *Mycobacterium tuberculosis* infection in humans and mice is the host’s ability to produce and respond to IFN-γ [1]–[4]. One well-characterized target gene of IFN-γ signalling is Indoleamine 2,3-dioxygenase-1 (IDO-1), the first rate-limiting enzyme of tryptophan metabolism along the kynurenine pathway, leading to the formation of tryptophan metabolites, including quinolinic and picolinic acid.

IDO-1 activity and products of the tryptophan-kynurenine pathway can influence immune functions through the effects of tryptophan depletion and the generation of biologically active tryptophan metabolites. IDO-1 activation has been shown to restrict intracellular growth of *Toxoplasma gondii*, *Leishmania donovanii* and *Chlamydia spec* by limiting tryptophan availability [5]–[7]. Similarly, IDO-1 activity in antigen-presenting cells (APCs) inhibits T cell proliferation, which can be reversed by the addition of excess tryptophan [8]. This indicates that IDO-1 activation may represent a means of regulating local T cell responses. Besides their effects on host immune cells, tryptophan metabolites can directly inhibit the growth of some bacterial pathogens in liquid cultures *in vitro* [9], [10].

IDO-1 expression and activation of the tryptophan-kynurenine pathway have been associated with mycobacterial infection in both humans and mice. Elevated levels of IDO-1 mRNA expression were detectable in sputum samples in a cohort of tuberculosis patients [11]. In mice, infection with *Mycobacterium bovis*, Bacille Calmette Guerin (BCG) lead to increased IDO-1 activity as indicated by elevated levels of kynurenine in serum, lung and brain [12]. In *M. tuberculosis*-infected mice, non-haematopoietic cells express IDO-1 during the chronic phase of infection. IFN-γ-receptor-deficiency in these cells lead to a lack of IDO-1 expression and was associated with exuberant neutrophil recruitment and increased mortality [13]. It is currently unknown if and how IDO-1 activity directly contributes to control of mycobacterial infection in the infected host. We hypothesized that IDO-1 could play a dichotomous role in the immune control of *M. tuberculosis*. Local tryptophan depletion as well as production of tryptophan metabolites might impair effective anti-mycobacterial T cell activation, differentiation and proliferation and, thus, favour survival and persistence of the pathogen. On the other hand, tryptophan metabolites have been shown to exert direct anti-mycobacterial effects, however at relatively high concentrations [13], [51]. We addressed the role of IDO-1 in the immune control of mycobacterial infection using human and murine macrophages as well as mice lacking IDO-1. Our data demonstrate that IDO-1 is one of the most highly induced genes in human macrophages infected with *M. tuberculosis*. Genetic ablation of IDO-1 expression or chemical inhibition of IDO activity did not affect intracellular survival of *M. tuberculosis* in cultured macrophages nor did it influence bacterial growth, T cell activation *in vivo* or survival of infected mice. Therefore, although *M. tuberculosis* induces expression of IDO-1 and subsequent activation of tryptophan metabolism, the lack of IDO-1 does not impact on the survival of the host or the pathogen in a mouse model of tuberculosis infection.

## Materials and Methods

### Human and Animal Ethics Statement

All mouse procedures performed in this study were conducted following the NIH guidelines for housing and care of laboratory animals and performed in accordance with institutional regulations after protocol review and approval by the University of Sydney Animal Ethics Committee (K75/2-2006/3/4281) and the Institutional Animal Care and Use Committee of Weill Cornell Medical College (#0607-511A).

Ethical clearance to obtain human broncho-alveolar lavage cells for this study was given by the Weill Cornell Medical College Review Board (#109005117) and all participants gave written informed consent. Experiments involving Buffy coats (obtained from the Red Cross, Approval # 05-06NSW-04, without any identifying information) were carried out with approval from the Sydney South West Area Health Service Human Research Ethics Committee (#X05-0178).

### Bacteria

*M. tuberculosis* (strain 1254 [ATCC 51910]), was purchased from American Type Culture Collection (ATCC). *M. tuberculosis* H37Rv and Erdman were obtained from R. North (Trudeau Institute, NY) and J.D. McKinney (EPFL, Lausanne, Switzerland), respectively.

### Animals

Female C57BL/6 mice at 6–10 weeks of age were purchased from the Animal Resource Centre (Perth, Australia) and Jackson Laboratory. IDO-1 gene deficient mice (IDO-1^−/−^) [14] were kindly provided by Prof. A. Mellor (Georgia Health Sciences University, GA). P25 CD4^+^ TCR transgenic mice (specific for residues 240–254 of Ag85B) [15] were kindly provided by Prof. K. Takatsu (University of Tokyo, Japan) and Prof. J. Ernst (New York University, NY). Mice were kept in specific pathogen-free conditions.

### Bacterial Cultures

Cultures of *M. tuberculosis* 1254, were grown until early log phase in Middlebrook 7H9 medium and then diluted to an optical density (OD) at 580 nm of 0.01. Picolinic acid, quinolinic acid, L-kynurenine, nicotinic acid, (all Sigma) or HEPES buffer (solvent control) were added. Bacterial growth was monitored in 150 µl aliquots in 96 well plates by OD_580 nm_ measurement. For survival in the presence of picolinic acid under different pH conditions, single-cell suspensions of *M. tuberculosis* Erdman cultures [16] were diluted to an OD_580 nm_ of 0.1 and exposed to picolinic acid and HEPES or MES as solvent controls, respectively, at 37**°**C in 7H9 medium at pH6.6 and pH5.5 for the times indicated. Serial dilutions of culture material were plated on Middlebrook 7H11 plates supplemented with 10% oleic acid/albumin/dextrose catalase (OADC) and 0.5% glycerol. Plates were incubated at 37**°**C for 3 weeks and bacterial numbers determined by enumerating colony forming units (CFU).

### Macrophage Cultures

Human broncho-alveolar lavage (BAL) cells from healthy individuals (macrophage content >90%) were plated in antibiotic free medium with IFN-γ (100 IU/ml) overnight. Cells were infected with *M. tuberculosis* 1254 for 4 h at a multiplicity of infection (MOI) of 1 and washed to remove extracellular bacteria. Bacterial numbers were determined by plating serial dilutions of macrophage lysates and CFU determination. Mononuclear cells were isolated from Buffy coats and differentiated into monocyte-derived macrophages (MDM) for 6 days in complete medium as previously described [17]. Macrophages were detached and plated in 96 well plates at 1 x 10^5^ cells/well in antibiotic free medium with IFN-γ (100 IU/ml) overnight, prior to mycobacterial infection. IFN-γ-activated macrophages were infected with *M. tuberculosis* H37Rv or *M. bovis* BCG Pasteur for 4 h at an MOI of 1 (Mtb) or 5 (BCG) and intracellular bacterial survival was monitored. Bone marrow cells from 8 to 10 week-old mice were differentiated into macrophages, and infected with *M. tuberculosis* 1254 or H37Rv from early log phase cultures at an MOI of 1. Intracellular survival was monitored as described [18].

### Gene Expression Analyses

For microarrays, human BAL cells were obtained from 4 individual donors and infected with *M. tuberculosis* 1254 at an MOI of 3–5. After 24 h, macrophages were lysed in Trizol (GIBCO BRL) and total RNA was isolated. After treatment with DNase I (Ambion) and purification (QIAGEN RNeasy), RNA (2–3 µg) was reverse transcribed (Superscript II; GIBCO BRL) with a T7-polyT primer and cDNA transcribed in the presence of biotinylated UTP and CTP (Enzo). Hybridization to Affymetrix GeneChip oligonucleotide arrays and scanning (Gene-Array Scanner) followed Affymetrix, Inc. protocols. Primary image analysis of the arrays was performed using GeneChip Microarray Analysis Suite 5.0 (Affymetrix, Santa Clara, CA) and images were scaled to an average hybridization intensity (average difference) of 250. Data analysis was carried out using GeneSpring 7.2 software (Silicon Genetics, Redwood City, CA). Normalization was applied using the distribution of all genes on each chip, i.e. all measurements on each chip were divided by the 50^th^ percentile value of that chip. Next, each gene was compared to its control by dividing its intensity by the average intensity of that gene in the untreated control macrophages. Data from 4 independent replicate experiments were used to perform a Wilcoxon two-sample rank test for each gene. Only genes with an Affymetrix “present call” in one of the two conditions compared were included in the analysis. A gene was considered “regulated” compared to control if its expression changed across the 4 experiments with P ≤ 0.05. Data mining was carried out with FileMakerPro 6.

For RT-PCR and quantitative RT-PCR total RNA was prepared from stimulated macrophages using RNAzol tri-reagent (Sigma) and transcribed into cDNA. PCR was performed using the gene specific primers shown in Table 1.

**Table 1:** Gene specific primers used in this study.

Gene	Forward 5′-3′	Reverse
hIDO-1	GGCAAAGGTCATGGAGATGT	TCCAGTTTGCCAAGACACAG
hGAPDH	CATGTTCGTCATGGGTGTGAA	ATGGACTGTGGTCATGAGTCCTT
mIDO-1	TTATGCAGACTGTGTCCTGGCAAA	TTTCCAGCCAGACAGATATATGCG
mb2m	TGACCGGCTTGTATGCTATC	CAGTGTGAGCCAGGATATAG
IDO-2	TGCCTGATGGCCTATAACCAGTGT	TGCAGGATGTGAACCTCTAACGCT
TDO	ATGAGTGGGTGCCCGTTTG	GGCTCTGTTTACACCAGTTTGAG

### Western Blot

Equal amounts of hMDM lysates were separated by SDS-PAGE and transferred onto nitrocellulose membrane. Briefly, lysates were loaded onto 0.75-mm, 12% polyacrylamide precast gels (Bio-Rad, Hercules, CA) and electrophoresed under denaturing non-reducing conditions. Proteins from the gels were transferred onto nitrocellulose membranes using a Bio-Rad Mini Transblot system for 1 h at 140 V. The membranes were blocked with PBS/5% skim milk powder overnight at 4°C, washed and incubated with sheep anti-human IDO-1 antibody (Chemicon International), followed by washing and incubation with rabbit anti-sheep IgG (Invitrogen), then donkey anti-rabbit IgG conjugated to HRP (Amersham, Life Science) all in PBS/1% BSA for 1 h at RT. Immunoreactive bands were detected by luminol detection kit (Pierce, Rockford, IL) after exposure to Kodak x-ray film using the Curix 60 X-ray developer (AGFA, Gevaert Ltd, Nunawading, Australia).

### Quantification of Tryptophan Metabolites

Human BAL cells (in 1% fetal bovine serum) were stimulated with *M. tuberculosis* 1254 (MOI 3) or human IFN-γ, (R&D Systems, 100 U or 500 U) for 48 h at 37**°**C. Human monocyte-derived macrophages were cultured for 24–72 h as described above. Quinolinic acid and picolinic acid were determined in cell culture supernatants using electron-capture negative-ion gas chromatography-mass spectrometry as described previously [19].

### In vitro T Cell Activation

Dendritic cells were generated from bone marrow as previously described [20]. DCs were cultured for 2 h with Ag85 peptide P25 before being overlaid with CFSE labelled CD4^+^ T cells from P25-specific T cell receptor transgenic mice [15]. T cell proliferation was determined by flow cytometry using a FACS LSRII (BD, San Jose, CA).

### Mouse Infections

Female C57BL/6 and IDO-1^−/−^ mice were infected with ∼100 cfu *M. tuberculosis* H37Rv by aerosol in a Middlebrook airborne infection apparatus (Glas-Col, Terre Haute, IN). Mycobacterial loads in infected organs were assessed by culture of serial dilutions of organ homogenates on 7H11 agar (Difco) at 37°C for 3 weeks.

To assess lymphocyte responses to mycobacterial antigens, single cell suspensions of lung and mediastinal lymph nodes were prepared as previously described [20], [21]. 2×10^5^ cells per well were incubated with culture filtrate protein (CFP) (kindly provided by Dr J. Belisle under the National Institute of Health TB research material and vaccine-testing contract NIH, NIAID Contract No. HHSN266200400091C) or at 1×10^5^ cells/well in ELISPOT plates overnight. The number of cells producing IFN-γ was determined as described previously [21].

To determine lung inflammation the percentage of the, formalin fixed, haematoxylin and eosin stained lung with inflammatory cell infiltration from WT and IDO-1^−/−^ mice was calculated using Image-Pro Plus software (Media Cybernetics, Silver Spring, USA) and Adobe Photoshop and is represented as the percentage of the whole lung tissue involved after subtracting the background cellularity in normal lung.

### Immunohistochemistry

Formalin-fixed paraffin-embedded sections were de-waxed in xylene, rehydrated through an ethanol gradient and washed in Tris-buffered saline (TBS) before blocking of endogenous peroxidase with 3% H_2_O_2_ in methanol. Sections were microwaved in 0.01 M citrate buffer (pH 6.0), for antigen retrieval, washed again in TBS then blocked with 20% normal horse serum for 30 min at RT. Sections were stained with polyclonal rabbit-anti-IDO-1 antibody (Enzo Life Sciences, USA) or rabbit control IgG (R&D Systems, USA) in a humidified chamber overnight at 4°C. Sections were washed in TBS before applying a HRP-conjugated horse-anti-rabbit IgG (Vector Laboratories, USA) for 30 min at RT, following which sections were stained with DAB peroxidase substrate (Vector Laboratories, USA) and counterstained with haematoxylin.

### Statistical Analysis

Statistical analysis was conducted by analysis of variance (Statview SAS Institute, NC). Fishers’ Protected Least Significant Difference analysis of variance post-hoc test was used for pair-wise comparison of data from multiple groups. Survival was calculated on a Kaplan-Meier nonparametric survival plot and significance was assessed by the Logrank-Mantel-Cox test. Differences with p<0.05 were considered significant.

## Results

### Mycobacteria-induced IDO-1 Expression in Macrophages

**Figure 1:**
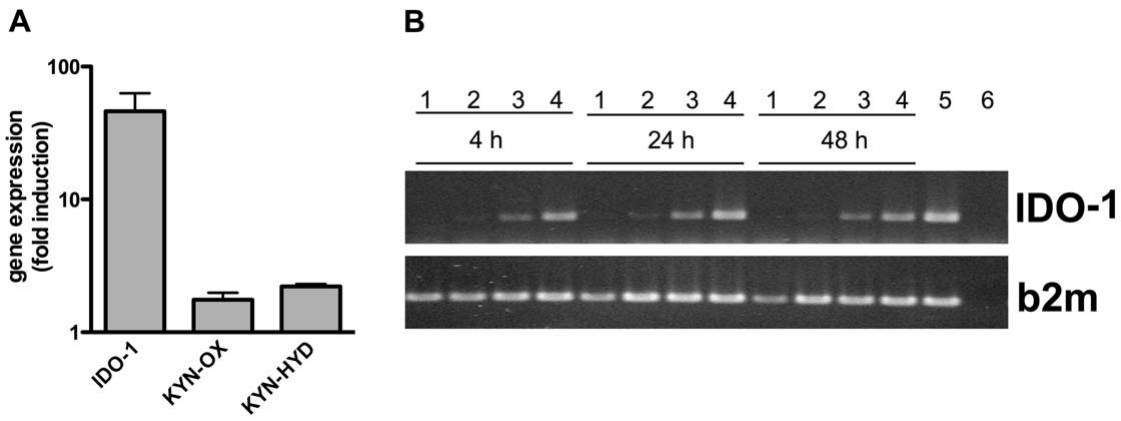
*M. tuberculosis* infection increases IDO-1 expression in human and murine macrophages. (A) Microarray results of BAL cells (>90% macrophages) infected with *M. tuberculosis* (MOI 3–5) for 24 h compared to uninfected cells. Increased mRNA expression levels for Indoleamine 2,3-dioxygenase (IDO-1), Kynurenine 3-monooxygenase (KYN-OX), and L-Kynurenine hydrolase (KYN-HYD) are depicted as fold induction upon *M. tuberculosis* infection compared to uninfected control cells. Data are means +/− standard error of comparisons of microarray data obtained from four different individuals. (B) Murine bone marrow-derived macrophages were left untreated (1), infected with *M. tuberculosis* (MOI 5) (2) and stimulated with IFN-γ (100 U/ml) (3). Co-stimulation with both *M. tuberculosis* and IFN-γ was either performed simultaneously (4) or cells were pre-treated with IFN-γ for 24 h and then infected with *M. tuberculosis* for 24 h (5) before RNA isolation. IDO-1 mRNA expression and beta-2-microglobulin (control) were detected at 4, 24, and 48 h by RT-PCR. (6) H_2_O. Data are representative of two independent experiments.

Human broncho-alveolar lavage (BAL) cells from four healthy individuals were infected with *M. tuberculosis* for 24 h and gene expression analyses were performed using microarrays. Compared to uninfected control cells, one of the genes most prominently upregulated in response to *M. tuberculosis* infection was IDO-1 (46.05 fold) (Fig. 1A). Furthermore, expression of two other enzymes in the kynurenine pathway, Kynurenine 3-monooxygenase and L-Kynurenine hydrolase, was enhanced in infected compared to uninfected cells at this time point (1.76 and 2.21 fold respectively) (Fig. 1A). IDO-1 mRNA and protein expression were also increased in IFN-γ-stimulated human monocyte-derived macrophages (hMDM) in response to infection with *M. bovis* BCG (Fig. S1A and S1B). Similarly, we observed an increase in IDO-1 mRNA expression in murine bone marrow-derived macrophages (mBMDM) upon infection with *M. tuberculosis*. IFN-γ stimulation in conjunction with *M. tuberculosis* further enhanced IDO-1 mRNA expression (Fig. 1B).

### The Tryptophan-kynurenine Pathway is Active in M. tuberculosis-infected Human Macrophages

**Figure 2:**
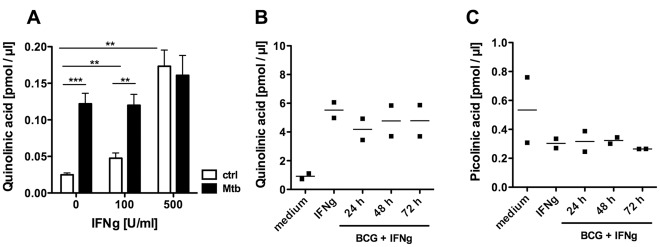
Mycobacteria and IFN-γ activate tryptophan-kynurenine metabolism in macrophages. (A) Human bronco-alveolar lavage cells were infected with *M. tuberculosis* (MOI 3) and stimulated with IFN-γ (100 and 500 U/ml) for 48 h. The concentration of quinolinic acid in cell culture supernatants was detected by electron-capture negative-ion gas chromatography-mass spectrometry. Data points are means +/− SD of triplicate wells representative of two independently performed experiments. Unpaired t test was used to compare raw data of three independent wells. (***P = 0.0003, **P<0.01, all other comparisons showed no significant difference) (B) Quinolinic and (C) picolinic acid concentrations in cell culture supernatants of human monocyte-derived macrophages stimulated overnight with IFN-γ (100 U/ml) before infection for 24, 48, and 72 h with *M. bovi*s BCG (MOI 5). Data points represent measurements obtained for cells from two individuals.

We next investigated if the tryptophan-kynurenine pathway is activated in *M. tuberculosis*-infected macrophages, by determining the concentrations of the tryptophan metabolites picolinic and quinolinic acid released from infected human alveolar macrophages and monocyte-derived macrophages (MDM). Upon infection of human alveolar macrophages with *M. tuberculosis* or stimulation with IFN-γ, the quinolinic acid concentration in the supernatant was increased (Fig. 2A). The simultaneous application of both *M. tuberculosis* and IFN-γ did not induce synergistic effects (Fig. 2A). Similarly, quinolinic acid concentrations were increased in cultures of MDM upon IFN-γ-stimulation compared to unstimulated controls (Fig. 2B). Subsequent infection of IFN-γ-primed MDM with mycobacteria did not further enhance the concentration of quinolinic acid in the supernatant over a period of three days following the infection (Fig. 2B). There was a considerable difference in the amounts of quinolinic acid produced by alveolar macrophages compared to MDM. This is in concordance with a previous observation showing differences in the production of quinolinic acid by different macrophage types (MDM compared to microglia), despite comparable levels of IDO-1 mRNA expression [22]. In contrast to quinolinic acid, no increased concentrations of picolinic acid were detected upon stimulation of MDM (Fig 2C).

### IDO-1-deficiency in Dendritic Cells Enhances Proliferation of Mycobacteria-specific CD4^+^ T Cells in vitro

**Figure 3:**
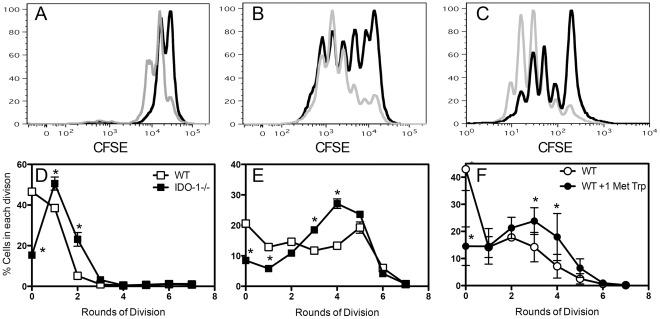
IDO-1 regulates CD4 T cell responses to mycobacterial antigen in vitro. Bone marrow-derived DCs were cultured for 4 days in vitro before stimulation for 2 h with Ag85 peptide P25. DCs were overlaid with CFSE labelled CD4^+^ T cells from P25-specific T cell receptor transgenic mice. Proliferation of T cells was determined by flow cytometry. DCs from WT and IDO-1^−/−^ mice were compared in their ability to induce proliferation of P25-specific T cells after 2 days (A, D) and 5 days (B, E). WT DCs were treated with 1 Methyl-DL-tryptophan before stimulation with P25 peptide. P25-specific T cell proliferation was determined after 5 days (C, F). (A-C) Fluorescence intensities of representative FACS plots are depicted. Grey- IDO-1^−/−^/1-Methyl-DL-tryptophan, Black – WT/untreated control. The percentages of cells per division were calculated (D-F). Data represent the mean +/− SD of triplicate cultures from one of three representative experiments. The differences between groups were analysed by analysis of variance, *P<0.001.

Along with modulation of macrophage functions, IDO-1 activity and tryptophan depletion have also been shown to suppress T cell proliferation [23]. We examined this in the context of a mycobacterial infection measuring the capacity of DCs from WT and IDO-1^−/−^ mice to induce the proliferation of P25-transgenic T cells, which recognise P25, a peptide from the mycobacterial secreted protein Ag85 [15]. In the absence of IDO-1 expression by DCs, T cells commenced proliferating earlier (Fig. 3A, 3D) and more cells had entered division by day 5 (Fig. 3B, 3E). Treatment of WT DCs with the IDO inhibitor 1-Methyl-DL-tryptophan [24] had a similar effect, with more cells entering division and undergoing more rounds of division than T cells in cultures with untreated DCs (Fig. 3C, F). Our findings support the hypothesis that IDO-1 activity in APCs can regulate T cell proliferation in response to mycobacterial antigens *in vitro*.

### Normal Cellular Influx and Generation of Antigen-specific T Cells in the Absence of IDO-1 During M. tuberculosis Infection in vivo

**Figure 4:**
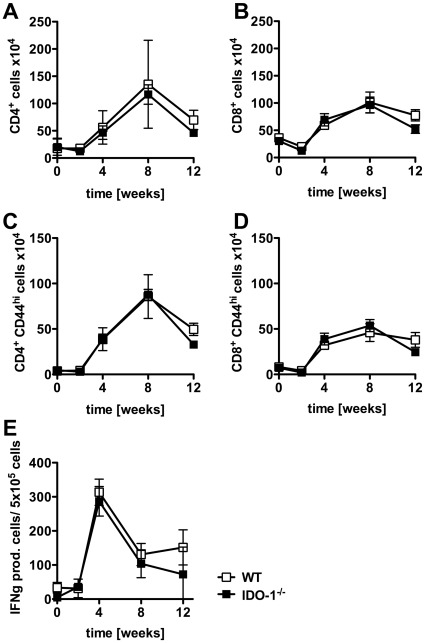
Normal cellular influx and the generation of antigen-specific T cells in the absence of IDO-1 in vivo. WT and IDO-1^−/−^ mice were infected with *M. tuberculosis* (100 CFU). Single cell suspensions were prepared from infected lungs and analyzed by flow cytometry for (A) CD4+, (B) CD8+ cells, and the percentages of these cells with an activated CD44hi/CD62lo phenotype ((C) CD4+, (D) CD8+). (E) Numbers of IFN-γ producing cells determined by ELISpot for lung cells cultured at 1×10^5^ cells/well with *M. tuberculosis* CFP overnight. Data represent the mean +/− SD of 5 mice from one representative of two experiments.

Given that IDO-1 deficiency enhanced mycobacteria-specific T cell proliferation *in vitro* we examined the influence of IDO-1 on the generation of an antigen-specific T cell response following infection with *M. tuberculosis in vivo*. WT and IDO-1^−/−^ mice were infected with *M. tuberculosis* via aerosol and the influx and activation status of CD4^+^ and CD8^+^ T cells in infected lungs were assessed over time. Similar total numbers of activated (CD44^hi^/CD62^lo^) CD4^+^ and CD8^+^ T cells were found in the lungs and lymph nodes of infected WT and IDO-1^−/−^ mice (Fig. 4A–D). Furthermore, the numbers of antigen specific T cells that produced IFN-γ in response to secreted *M. tuberculosis* antigens were equivalent in WT and IDO-1^−/−^ mice throughout the course of infection (Fig. 4E). Thus, IDO-1-deficiency did not affect the influx or activation of *M. tuberculosis*-specific T cells *in vivo*.

### IDO-1 is not Essential to Control M. tuberculosis Growth in the Host or Enhance Survival of Infected Mice

**Figure 5:**
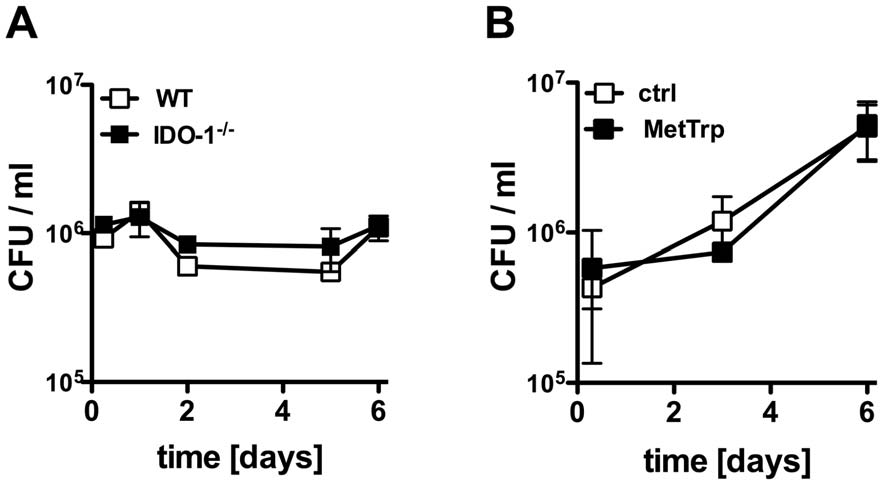
Control of M. tuberculosis infection by macrophages in the absence of IDO-1 activity. (A) Bone marrow-derived macrophages of WT and IDO-1^−/−^ mice were infected with *M. tuberculosis* (MOI 1) in the presence of IFN-γ (100 U/ml). Intracellular bacterial numbers were determined at the indicated time points. Data points are means +/− SD of triplicate wells of one representative of 3 independent experiments. (B) Human BAL cells were stimulated with 100 U/ml IFN-γ and infected with *M. tuberculosis* (MOI 1) in the presence or absence of 1-Methyl-DL-Tryptophan (250 µM). Bacterial counts in cell lysates were determined at the times indicated. Data points are means +/− SD of triplicate wells of one representative of 6 independent experiments.

We further investigated if IDO-1 activity in macrophages modulated intracellular survival of *M. tuberculosis*. To this end, we infected WT and IDO-1^−/−^ murine macrophages with *M. tuberculosis* and monitored intracellular bacterial numbers over time. Similar bacterial numbers were observed in IDO-1^−/−^ and WT macrophages (Fig. 5A). Furthermore, when infecting human or murine macrophages with *M. tuberculosis* in the presence or absence of the IDO inhibitor 1-Methyl-DL-tryptophan [24], no differences in intracellular bacterial numbers were detected (Fig. 5B and Fig. S2).

**Figure 6:**
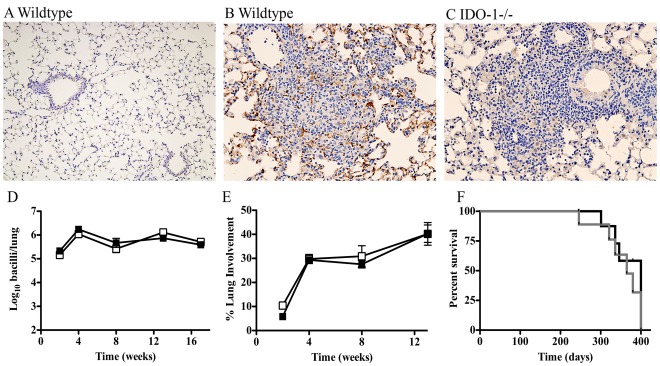
IDO-1 is dispensable for the control of M. tuberculosis infection in vivo. WT and IDO-1^−/−^ mice were infected with *M. tuberculosis* (100 CFU). IDO-1 expression in the lungs of uninfected WT mice (A), and WT (B) and IDO-1^−/−^ (C) mice at 28 days post infection. Lungs from uninfected IDO-1^−/−^ mice showed no IDO-1-positive cells (data not shown). IDO-1-expression in lungs of WT mice was primarily associated with inflammatory lesions within large monocytic cells (B), while IDO-1^−/−^ mice show no specific staining (C). One representative section from 1 of 5 mice per group. Bacterial loads (D) in infected lungs were determined at the time points indicated. Data represent the means +/− SD of 5 mice per group from one of two independent experiments. Lung inflammation (E) as percentage of the lung area with inflammatory cell infiltration from WT and IDO-1^−/−^ mice after subtracting the background cellularity in normal lung. To assess survival (F), infected mice were monitored daily and euthanized when showing signs of ill health. Data represent the time to euthanasia of 6 mice per group.

Although the absence of IDO-1 did not influence the development of an antigen-specific T cell response *in vivo*, we found that IDO-1 is strongly expressed within the lungs of *M. tuberculosis*-infected mice (Fig. 6A–C). Thus, we examined the requirement for IDO-1 to control bacterial growth, inflammation, and survival of mice infected with *M. tuberculosis*. Bacterial burden in WT and IDO-1^−/−^ mice in lungs, spleen, and livers were similar during the course of infection (Fig. 6D and Fig. S3). The extent and quality of the inflammatory infiltrate in lungs of IDO-1 deficient mice were comparable to WT mice (Fig. 6E) and equivalent survival post infection was observed in both WT and IDO-1^−/−^ mice (Fig. 6F). These results demonstrate that IDO-1 was not essential for the immune control of *M. tuberculosis in vivo* or the survival of infected mice.

### Inhibition of Mycobacterial Growth by Picolinic Acid in vitro

**Figure 7:**
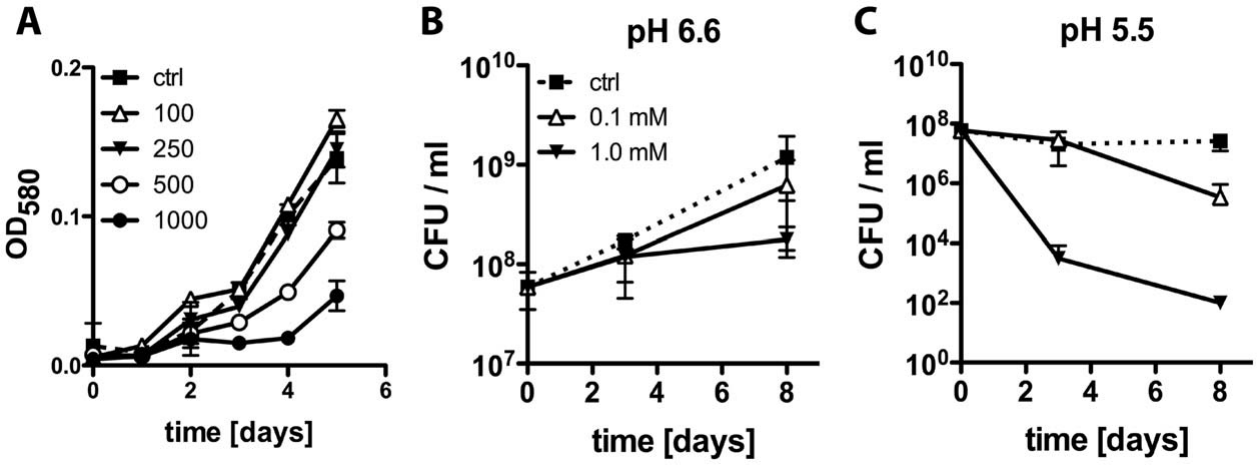
Inhibition of mycobacterial growth in vitro by picolinic acid. (A) *M. tuberculosis* was grown in 7H9 liquid medium in the presence of picolinic acid (100 - 1000 µM). Optical density of cultures was measured. Data are the mean +/− SD of four independent wells per condition and are representative of 2–4 independent experiments. *M. tuberculosis* CFU were determined upon exposure to picolinic acid (0.1 and 1 mM; HEPES and MES as buffer controls) in 7H9 liquid medium at pH 6.6 (B) and 5.5 (C). Data represent means +/− SD of triplicate wells. Similar pH-dependent effects of picolinic acid on *M. tuberculosis* survival were observed in three independent experiments.

*M. tuberculosis* infection of macrophages led to IDO-1 expression and production of quinolinic acid, but not picolinic acid. Therefore, the direct effect of tryptophan metabolites on *M. tuberculosis* growth in liquid culture was examined. Neither quinolinic acid nor kynurenine, an upstream tryptophan metabolite, inhibited growth of *M. tuberculosis* (Fig. S4). Whereas picolinic acid, the metabolite not produced by *M. tuberculosis* infected macrophages (Fig. 2C), inhibited *M. tuberculosis* growth in a dose dependent fashion at high µM concentrations (Fig. 7A). However, tryptophan metabolite concentrations in biological samples, such as serum and cerebrospinal fluid in humans and mice, are in the low µM range, reaching up to 100 µM in severe malaria [25]–[27]. We determined if lower concentrations of picolinic acid could act synergistically with known antimicrobial effector mechanisms in macrophages, such as acidification of the phagosome. At pH 6.6, picolinic acid was bacteriostatic, whereas at pH 5.5 picolinic acid exhibited bactericidal activity (Fig. 7B, 7C). Furthermore, while at pH 6.6 100 µM picolinic acid did not impair *M. tuberculosis* growth after 8 days of exposure, the same concentration of picolinic acid at pH 5.5 had bactericidal activity resulting in a 78-fold decrease of viable *M. tuberculosis* in the medium. These findings suggest that tryptophan metabolism in infected macrophages bears the potential to generate anti-mycobacterial compounds if directed towards the production of picolinic acid in an acidified compartment.

## Discussion

The roles of IDO-1 and tryptophan metabolism as regulatory components of the immune system have gained much attention in recent years. Here we demonstrate that *M. tuberculosis* infection upregulated IDO-1 expression in both human and murine macrophages *in vitro* and in inflammatory foci and surrounding tissue in lungs of *M. tuberculosis*-infected mice (Fig. 1, S1, 6A–C). IDO-1 expression in infected mouse lungs has been reported [13], and elevated IDO-1 expression has recently been correlated with the expression of other inflammatory markers, including C-reactive protein and poor prognosis of tuberculosis patients [28]. With our study we therefore aimed to address the functions of IDO-1 during *M. tuberculosis* infection.

Tryptophan metabolism has been extensively studied as a means of regulating T cell functions in settings as diverse as tumor-induced immune escape, peripheral tolerance and inflammation during infection [29]–[31]. Enhanced IDO-1 activity during bacterial infection might limit tryptophan availability to T cells and thereby regulate T cell proliferation [32]. Our *in vitro* studies indeed showed that IDO-1 activity in APCs inhibited mycobacterial antigen-specific T cell proliferation. Of note, recent studies by Li *et al.* suggest that pleurisy fluid from tuberculosis patients contains inhibitory components that interfere with T cell responses and that this can be partially blocked with the IDO-inhibitor, 1-Methyl-DL-tryptophan [33], [34]. Together these observations strongly suggested a role for IDO-1 activity in inhibiting *M. tuberculosis*-specific adaptive immune responses and, thereby, potentially aiding the persistence of this pathogen within the infected host. However, our data demonstrate that IDO-1-deficiency *in vivo* did not increase the number of IFN-γ-mycobacteria-specific T cells nor did it alter the numbers of activated T cells in the lungs and lymph nodes of *M. tuberculosis*-infected mice. Furthermore, IDO-1^−/−^ mice did not show altered inflammatory responses as demonstrated by the similar numbers, size and composition of lesions in infected lungs. Increased liver inflammation in IDO-1^−/−^ mice infected with *Rhodococcus equi* [35] has been reported, suggesting that the involvement of IDO-1 in modulating inflammation in infected organs my be dependent upon the infecting pathogen.

IDO-1 activity has also been implicated in modulating Th1 and Th17 responses during infection with *Candida albicans* and the balance between Th17 and regulatory T cell responses in HIV disease [36], [37]. A recent study suggested the presence of an immune-regulatory loop mediated by IFN-γ-induced IDO-1 expression in non-hematopoietic cells that controlled IL-17 responses and neutrophil influx into infected lungs, which was correlated to the survival of mice during *M. tuberculosis* infection [13]. Pathogenic neutrophilia in tuberculosis has further been suggested to be associated with a failed Th1 response [38]. We have previously shown that Th17 cells can confer some protection to *M. tuberculosis* infection in mice in the absence of IFN-γ producing Th1 cells, and this is accompanied by increased inflammation and damaging pathology [39]. The role of regulatory T cells (Tregs) in pulmonary tuberculosis is not fully understood but functions for Tregs in controlling bacterial burden in the lung as well as the frequency of IFN-γ producing cells have been reported [40], [41], [42]. If Th17/IL-17 or Treg numbers and functions during *M. tuberculosis* infection had been markedly altered by the deficiency in IDO-1, we would have expected this to lead to altered T cell activation, affect lung inflammation and alter the bacterial burden in infected organs. As none of these phenotypes were observed in IDO-1−/− mice during *M. tuberculosis* infection, this suggests that any modulation of Th17 or Treg responses that occurred in the absence of IDO-1 did not significantly diminish the capacity of IDO-1^−/−^ mice to control the disease.

Our *in vitro* data clearly demonstrate that DCs from IDO-1^−/−^ mice, or DCs treated with 1-Methyl-DL-tryptophan, facilitated more rapid T cell proliferation in response to a mycobacterial antigen, in concordance with previous reports describing IDO-1-mediated inhibition of T cell proliferation *in vitro* [8], [43]. In contrast, we observed no modification of anti-mycobacterial responses *in vivo*. The different results observed in *in vitro* and *in vivo* models might reflect the different microenvironments of isolated cell culture systems versus complex organs as well as different immunological requirements to control acute versus chronic T cell mediated immune responses. Furthermore, compensatory mechanisms may mask the impact of IDO-1 activity on maintaining effective T cell mediated immunity against *M. tuberculosis in vivo*. There are two other tryptophan-metabolising enzymes, IDO-2 and TDO (tryptophan dioxygenase), that could drive the kynurenine pathway in the absence of IDO-1, but their role in immunity to infection is not well understood. TDO activity is mainly found in the liver, while IDO-2 is expressed in the kidney, liver, epididymis and testis, but not the lung [44], [45]. We could not detect IDO-2 expression in the lungs of uninfected C57BL/6 wild type and IDO-1^−/−^ mice or during *M. tuberculosis* infection in C57BL/6 mice (data not shown). We further found that TDO was only expressed at very low levels in mouse lungs and expression was not upregulated by *M. tuberculosis* infection (data not shown), suggesting that neither TDO nor IDO-2 contribute significantly to the control of *M. tuberculosis* infection in the lung. Tissue expression of both IDO-2 and TDO has previously been shown to be independent of IFN-γ. IDO-2 remained unchanged during *Plasmodium berghei* ANKA infection [46] and TDO activity has also not been associated with immune-related tryptophan metabolism [47]. Taken together, these observations make it seem unlikely that either enzyme compensates for the absence of IDO-1 activity during *M. tuberculosis* infection.

For some pathogens tryptophan availability within the infected host might be a limiting factor rendering them susceptible to tryptophan depletion by IDO-1 activity [32]. A *trpD* mutant *M. tuberculosis* strain has been described to be auxotroph for tryptophan [48] and it is possible that *M. tuberculosis* can synthesize tryptophan within the infected host. When we examined the anti-microbial effects of individual tryptophan metabolites our data demonstrated that quinolinic acid and L-kynurenine did not affect growth of *M. tuberculosis,* whereas picolinic acid strongly inhibited the growth of several mycobacterial species, but had minimal impact on gram-positive and no effect on gram-negative bacteria (Fig. 7 and data not shown).

Picolinic acid is an effective activator of macrophages [49] and decreased intracellular replication of *M. avium* in murine macrophages [50]. While the underlying mechanism is unclear, iron availability [51], interference with phago-lysosme interactions and induction of host cell apoptosis [50], [52] have been suggested. Elevated levels of picolinic acid have been associated with cerebral malaria in patients [25] and were detected in a mouse model of malaria infection [53]. The picolinic acid concentrations required for growth inhibition of *M. tuberculosis in vitro*, however, are at least 10–100 times higher that those observed in serum, cerebrospinal fluid or infected macrophage cell cultures [25]–[27] (Fig. 2). However, our data show that picolinic acid and an acidified environment could provide an efficient combination of anti-mycobacterial effector mechanisms. Yet, this would require the presence of both in the same compartment. As we could not detect picolinic acid in the supernatants of macrophage cultures upon mycobacterial infection or IFN-γ activation (Fig. 2C), the absence of this potent anti-microbial agent from activated macrophages might render this host cell an attractive niche for intracellular pathogens like *M. tuberculosis*.

It is clear from the growing literature surrounding the many functions of IDO-1 that our understanding of the impact of tryptophan metabolism on immune regulation is far from complete. The activation of tryptophan metabolism appears to be part of the anti-microbial repertoire against some but not all pathogenic bacteria. Growing evidence also indicates that IDO-1 activation and tryptophan metabolism in macrophages within the central nervous system is involved in AIDS-related dementia and other inflammatory brain conditions [45], [54]. Inhibition of IDO-1 activity is thus an attractive therapeutic strategy in treating HIV-associated neurological disorders. In light of the high prevalence of patients infected with both HIV and *M. tuberculosis* our findings indicate that therapeutic targeting of IDO-1 should not increase the risk of exacerbating tuberculosis in HIV patients.

In conclusion, we demonstrate that despite a robust increase in IDO-1 expression, activation of tryptophan metabolism in infected macrophages and a role for DC-derived IDO-1 in regulating T cell responses *in vitro*, immune control of *M. tuberculosis* in macrophages occurs independently of IDO-1 and IDO-1 is redundant for regulating control of *M. tuberculosis* infection in a mouse model of tuberculosis.

## Supporting Information

Figure S1 **Induction of IDO-1 expression by M. bovis BCG in human monocyte-derived macrophages.** Human monocyte-derived macrophages were stimulated with 100 U/ml IFN-γ and infected with *M. bovis BCG* (MOI 5). (A) IDO-1 mRNA expression was determined by qRT-PCR at 24 h post infection. Data points represent mean +/− SD of IDO-1 mRNA expression compared to uninfected control PBMC from 9 individuals. (B) IDO-1 protein expression in cells of two individuals, stimulated for 24–72 h as described above, was determined by Western Blot.
(TIF)

Figure S2 **1-Methly-DL-tryptophan does not affect the intracellular survival of** ***M. tuberculosis*** **in murine bone marrow-derived macrophages.** Bone marrow-derived macrophages of C57BL/6 mice were stimulated with IFN-γ (100 U/ml) were infected with *M. tuberculosis* (MOI 1) in the presence or absence of 1-Methyl-DL-tryptophan (250 µM). Intracellular bacterial numbers were determined at the indicated time points. Data points are means +/− SD of triplicate wells of one representative of three independent experiments.
(TIFF)

Figure S3 **Bacterial burden in spleens and livers of WT and IDO-1^−/−^ mice infected with** ***M. tuberculosis*** **.** Mice were infected *M. tuberculosis* (100 CFU) and bacterial loads in infected spleens (A) and livers (B) were determined over time. Data represent mean +/− SD of 5 mice per group from one of two independent experiments.
(TIFF)

Figure S4 **Growth of** ***M. tuberculosis*** **in the presence of tryptophan metabolites.** M. tuberculosis was grown in 7H9 liquid medium in the presence of picolinic acid and L-kynurenine and quinolinic acid (1 mM). Optical density of the cultures was measured at 580 nm. Data are means +/− SD of four independent wells per condition and are representative of 2–4 independent experiments.
(TIFF)
