# Supplementary figures and images for: M. tuberculosis Induces Potent Activation of IDO-1, but This Is Not Essential for the Immunological Control of Infection
Source: PLoS One. 2012 May 23;7(5):e37314. doi: 10.1371/journal.pone.0037314 (PMC3359358; doi:10.1371/journal.pone.0037314)

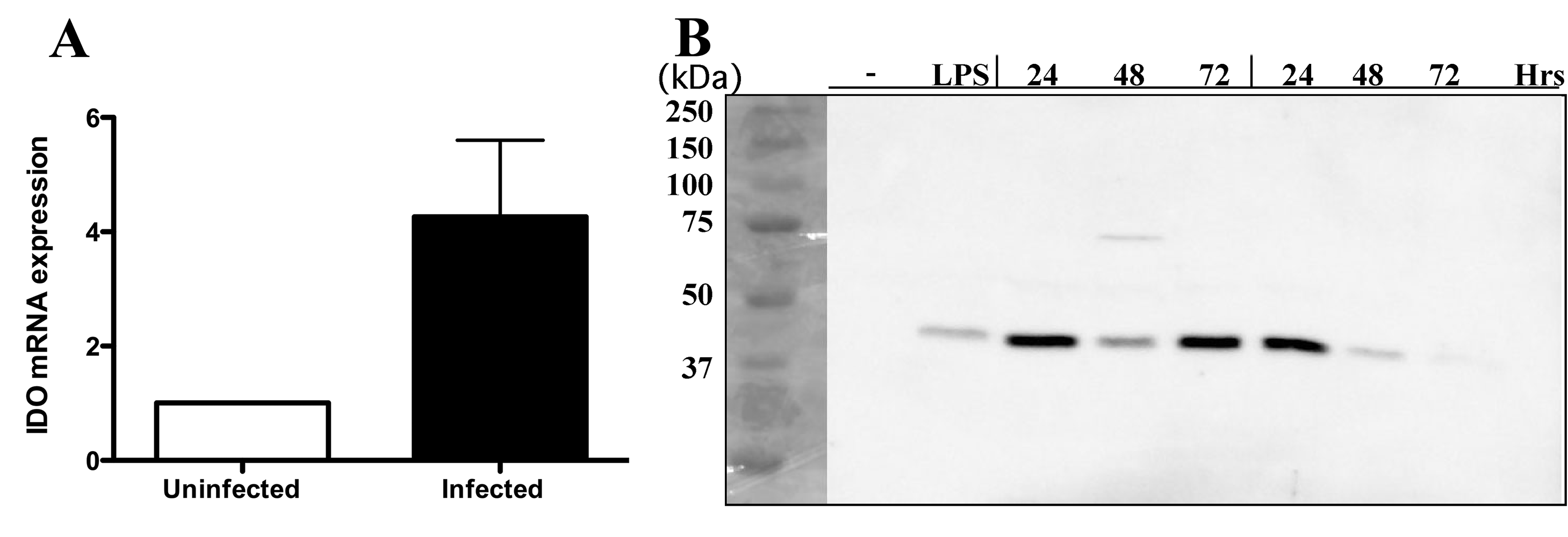

Supplement: Figure S1 — Induction of IDO-1 expression by M. bovis BCG in human monocyte-derived macrophages. Human monocyte-derived macrophages were stimulated with 100 U/ml IFN-γ and infected with M. bovis BCG (MOI 5). (A) IDO-1 mRNA expression was determined by qRT-PCR at 24 h post infection. Data points represent mean +/− SD of IDO-1 mRNA expression compared to uninfected control PBMC from 9 individuals. (B) IDO-1 protein expression in cells of two individuals, stimulated for 24–72 h as described above, was determined by Western Blot. (TIF) [file pone.0037314.s001.tif]

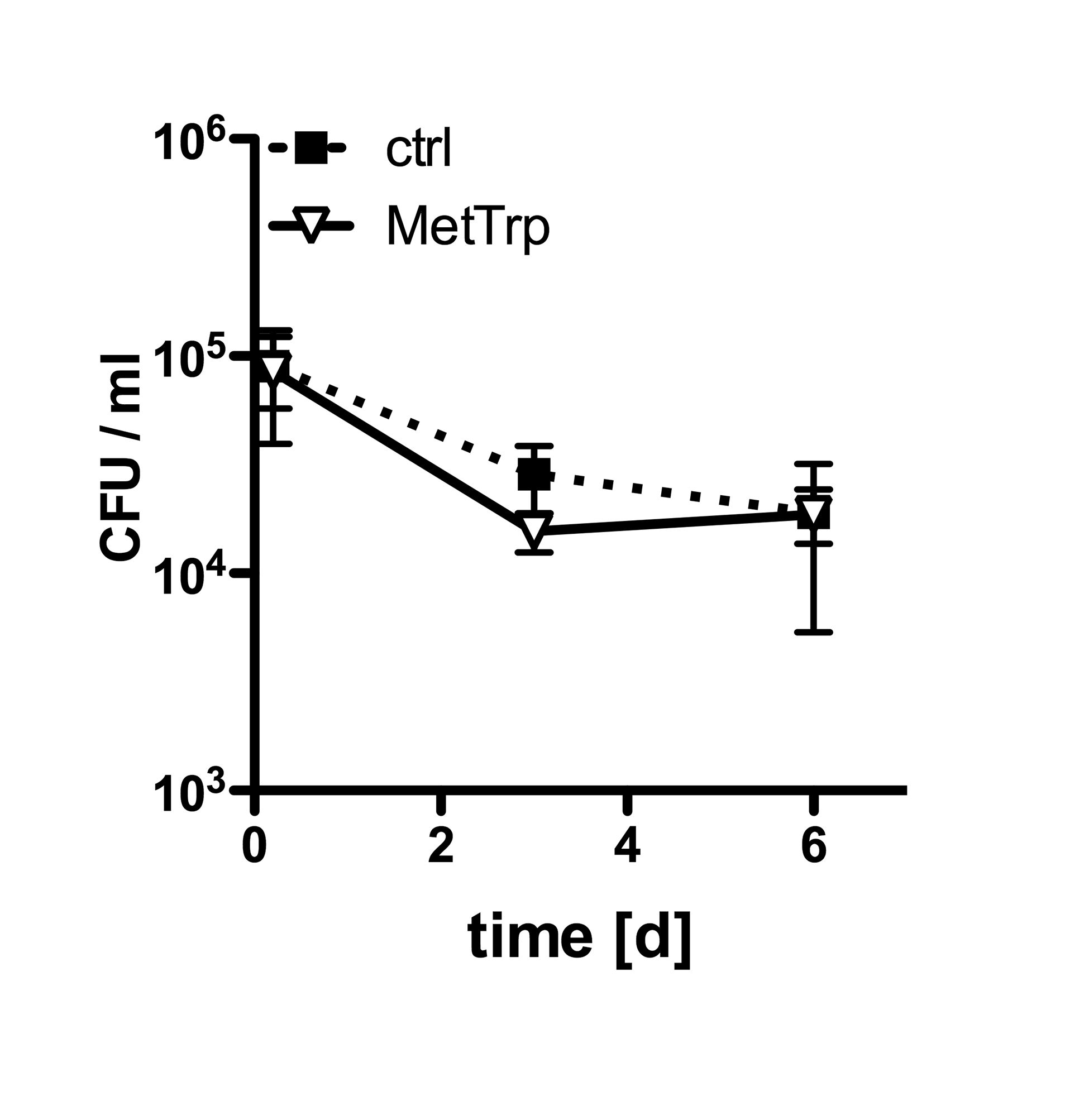

Supplement: Figure S2 — 1-Methly-DL-tryptophan does not affect the intracellular survival of M. tuberculosis in murine bone marrow-derived macrophages. Bone marrow-derived macrophages of C57BL/6 mice were stimulated with IFN-γ (100 U/ml) were infected with M. tuberculosis (MOI 1) in the presence or absence of 1-Methyl-DL-tryptophan (250 µM). Intracellular bacterial numbers were determined at the indicated time points. Data points are means +/− SD of triplicate wells of one representative of three independent experiments. (TIFF) [file pone.0037314.s002.tiff]

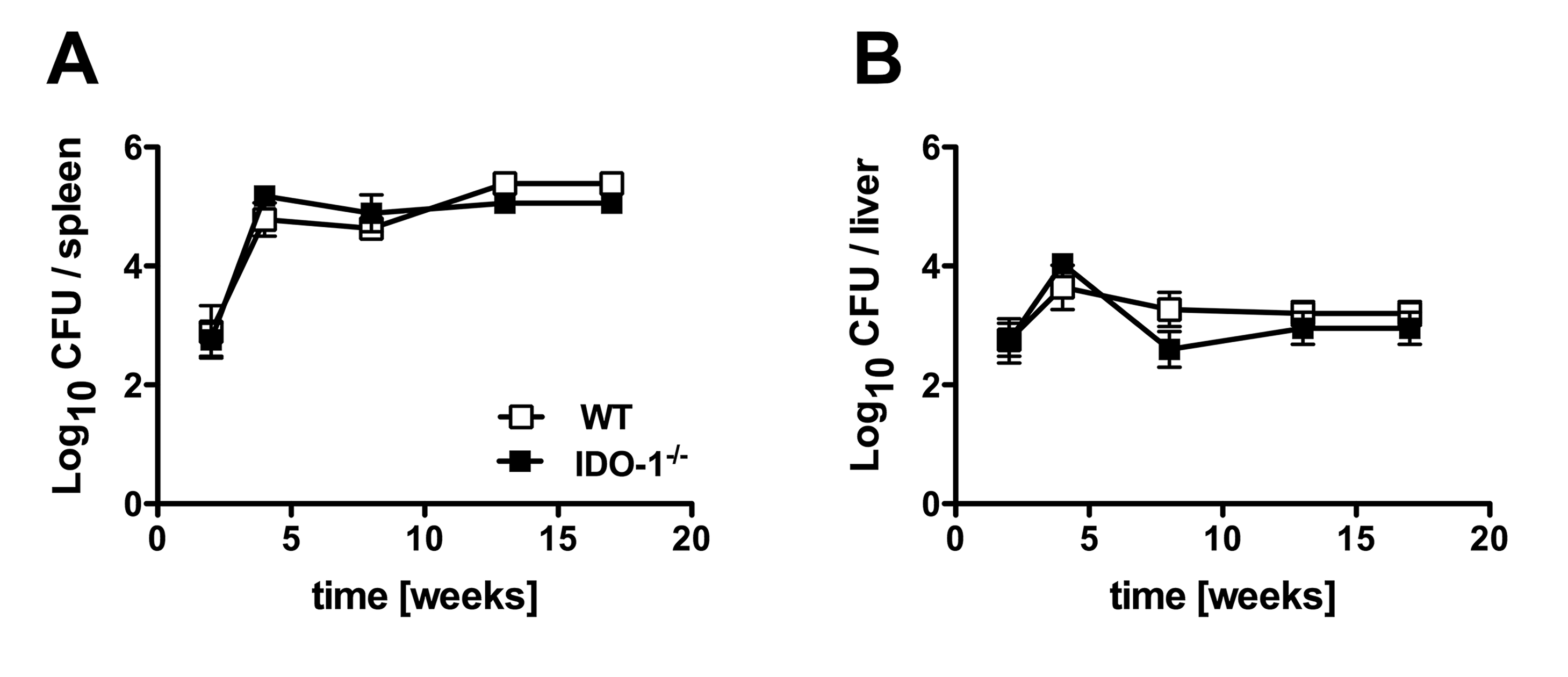

Supplement: Figure S3 — Bacterial burden in spleens and livers of WT and IDO-1−/− mice infected with M. tuberculosis . Mice were infected M. tuberculosis (100 CFU) and bacterial loads in infected spleens (A) and livers (B) were determined over time. Data represent mean +/− SD of 5 mice per group from one of two independent experiments. (TIFF) [file pone.0037314.s003.tiff]

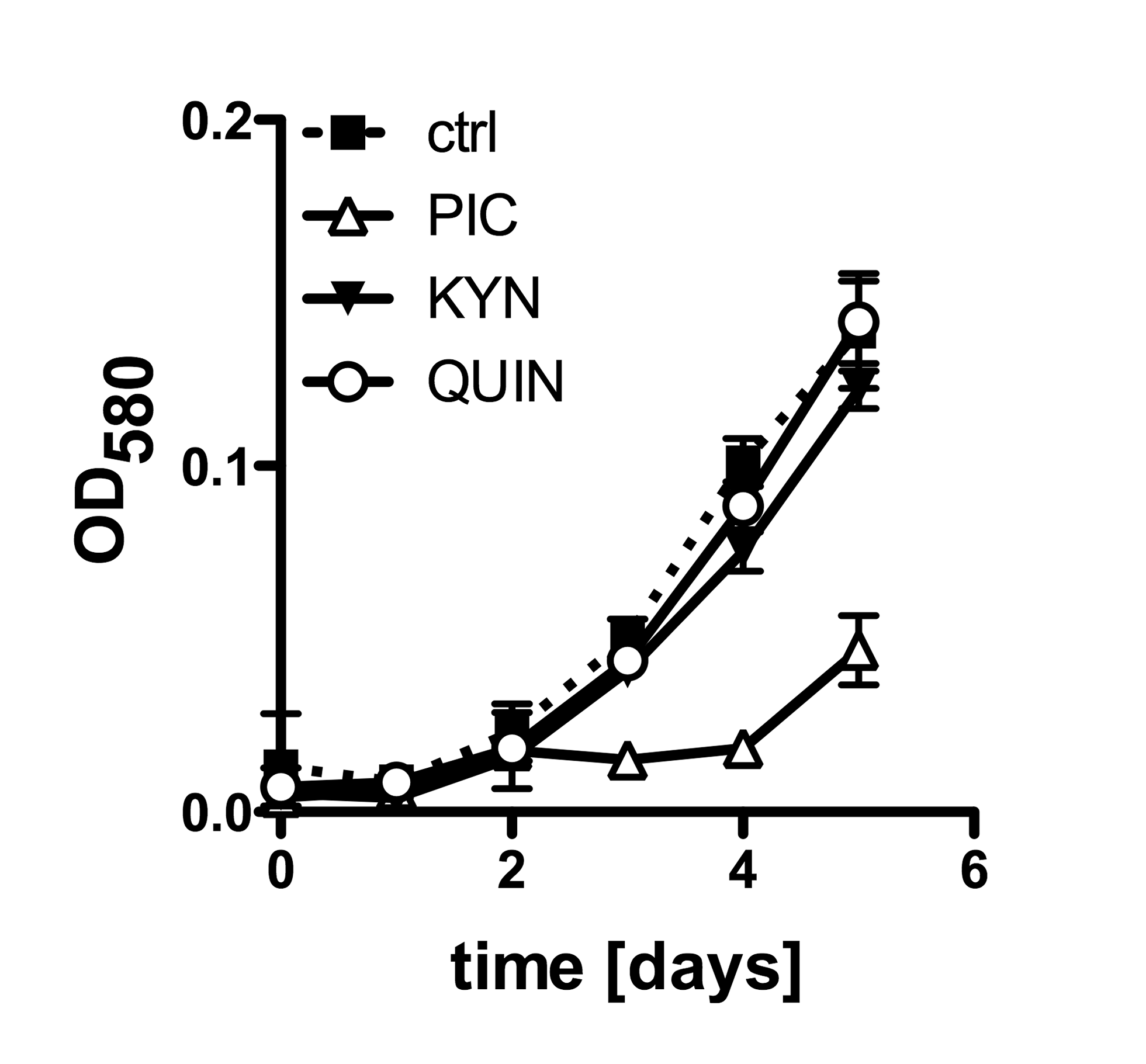

Supplement: Figure S4 — Growth of M. tuberculosis in the presence of tryptophan metabolites. M. tuberculosis was grown in 7H9 liquid medium in the presence of picolinic acid and L-kynurenine and quinolinic acid (1 mM). Optical density of the cultures was measured at 580 nm. Data are means +/− SD of four independent wells per condition and are representative of 2–4 independent experiments. (TIFF) [file pone.0037314.s004.tiff]
